# Multiphysical characterization for predicting compressive strength of Portland cement concrete using synthetic aperture radar, ultrasonic testing, and rebound hammer

**DOI:** 10.1038/s41598-024-83829-y

**Published:** 2025-02-19

**Authors:** Maryam Abazarsa, Tzuyang Yu

**Affiliations:** Department of Civil and Environmental Engineering, University of Massachusetts Lowell, Lowell, MA 01854 USA

**Keywords:** Multiphysical characterization, Synthetic aperture radar, Compressive strength, Concrete cylinders, Ultrasonic pulse velocity, Rebound hammer, Civil engineering, Electrical and electronic engineering

## Abstract

Portland cement concrete (PCC) is a versatile and widely used construction material renowned for its strength and durability. The mechanical properties of PCC, including compressive strength, flexural strength, and splitting tensile strength, play a pivotal role in ensuring the safety and sustainability of structures such as buildings, bridges, and dams. Traditionally, the determination of PCC’s compressive strength involves destructive testing of standard-size concrete cylinders until they fail. While nondestructive evaluation (NDE) techniques are available for assessing these properties, they often require direct contact between the sensor and the concrete surface, making them less efficient and practical compared to remote sensing techniques. In this paper, three NDE techniques were applied for estimating the mechanical properties of concrete, including synthetic aperture radar (SAR), ultrasonic testing (UT), and a rebound hammer (RH). A total of 48 laboratory concrete cylinders (diameter = 3", height = 6") were manufactured. These cylinders were created with different water-to-cement ratios (0.4, 0.45, 0.5, and 0.55) with a mix design ratio of 1:2:3 for cement: sand: gravel (by mass). Before these cylinders were tested by destructive compression test, they were measured by three NDE techniques. A 10 GHz SAR system, a 54 kHz UT system, and a RH sensor were used to inspect those cylinders at different concrete ages (7, 14, 28, and 96 days). From our result, the performance ranking among three NDE techniques was individually UT, SAR, and RH. When combining two NDE techniques, SAR with UT delivered the best performance. Multiphysical NDE (SAR with UT) outperformed uniphysical NDE (UT with RH) on the prediction of compressive strength of concrete, with a highest R^2^ value of 0.9918. This research demonstrates the promising potential of multiphysical NDE for other engineering problems.

## Introduction

Characteristics of Portland cement concrete such as low cost, high compressive strength, and good durability have made it the most-widely used construction material in the world. Among many properties of concrete, compressive strength is the most important property since the safety of concrete structures is essentially based on the compressive strength of concrete. Development of concrete’s compressive strength is a time-dependent, thermochemical process, depending on the mix design of concrete. In general, for most ordinary concrete designs using general purpose cement (Type I/II), the compressive strength development of concrete is a nonlinear process^1–3^ It increases exponentially at the early age and then logarithmically in the long-term depending on curing conditions^4^. The 28-day strength is customarily used for ordinary concrete by researchers and practitioners, as well as in codes and standards^5–7^. Destructive compression tests (e.g. ASTM C39/C39M^5^) on at least three specimens per concrete batch are required to address the statistical nature of aggregate distribution inside concrete. However, in field applications, destructive compression tests can only be applied to concrete cores extracted from limited locations on a real structure. Therefore, distribution of actual compressive strength remains unknown on most areas of a concrete structure.

To address this issue, the use of nondestructive testing/evaluation/inspection (NDT/E/I) techniques to determine the compressive strength of concrete in an in-situ configuration has been a promising solution, including ground penetrating radar (GPR)^8–10^, rebound hammer (RH), ultrasonic testing (UT)^11^, four-probe electrical resistivity method^12^, X-ray computed tomography (CT)^13^, piezoelectric sensors^14–16^, open-ended coaxial probes^17^, and synthetic aperture radar (SAR)^9,18^. The nondestructive nature of these techniques allows engineers to repeatedly monitor the development of concrete compressive strength at the same locations over time without causing additional concerns or needs for repair (e.g., patching). When using radar sensors (waveguides, GPR, and SAR) for NDT/E/I, it is materials’ electromagnetic properties (electrical and magnetic) that determine the inspection result. The electrical property of construction materials can be described by the relative complex electric permittivity whose real part is dielectric constant and imaginary part loss factor. The magnetic property of construction materials can be described by the relative complex magnetic permeability. When using electrical sensors (coaxial probes), only the electrical property of material is measured. On the other hand, mechanical sensors (RH, UT) interrogate materials’ mechanical properties (e.g., Young’s modulus, Poisson’s ratio) by measuring either ultrasonic pulse velocity (UPV) in UT or an empirical rebound value in RH to correlate with the compressive strength of concrete.

Both radar sensors and mechanical sensors have been applied individually to the determination of compressive strength of concrete in the literature. Abo-Qudais^11^ studied the effect of concrete aggregate gradation, the water–to-cement (w/c) ratio (0.4, 0.45, 0.5, and 0.55), and the curing time (3, 7, 28, and 90 days) on the UPV of concrete. They used 54 kHz frequency in their UPV measurement. From their result, the UPV of concrete was found to decrease with the increase of the w/c ratio of concrete. Larger aggregate sizes in concrete increased the measured UPV of concrete. In addition, the UPV of concrete increased with the increase of concrete curing time. Lai et al.^19^ applied a 1 GHz GPR system to study the relationship between compressive strength and dielectric constant of lightweight and normal aggregate concretes. They casted and cured concrete specimens to monitor the change of material properties (compressive strength, apparent porosity, saturated density) at different concrete ages (1, 4, 7, 28, 56 and 90 days). Their result indicated that the dielectric constant of concrete nonlinearly decreases with the increase of compressive strength of concrete. Ferreira and Jalali^20^ used a four-probe electrical method to predict the 28-day compressive strength of concrete cubes (6") and cylinders (4"-by-8" and 6"-by-12") for two water-to-cement (w/c) ratios (0.4 and 0.5) using the 7-day electrical resistivity measurements. They found that the electrical resistivity and the compressive strength of concrete are linearly correlated, with a R^2^ (coefficient of determination) value in the range of 0.94959 (w/c = 0.5 cylinders) and 0.98263 (w/c = 0.4 cylinders). The increase of the 28-day compressive strength was associated with the increase of the electrical resistivity of concrete. Shen et al.^21^ used 1.5 GHz GPR to correlate the compressive strength of concrete (w/c = 0.5) at different ages (1, 2, 3, 5, 7, 14 and 28 days) with the dielectric constant of concrete. Their experimental result indicates that the increase of compressive strength leads to the decrease of dielectric constant of concrete. Alzeyadi and Yu^18^ used a 10.5 GHz SAR imaging system and the K-R-I transform to study the effects of chloride content in oven-dried concrete specimens on SAR images. Six groups of different chloride contents (0%, 2%, 4%, 6%, 8% and 10% of cement weight) were cast and oven-dried to remove the effect of moisture (physically bound water). They found that the integrated SAR amplitude and the average maximum SAR amplitude increase nonlinearly with the increase of the moisture content in concrete, as the moisture content in concrete reduces the compressive strength of concrete. Yu et al.^9^ applied a 10 GHz SAR imaging system to monitor the strength and moisture content and distribution inside the ultra-high-performance concrete (UHPC). They obtained both integrated and the maximum SAR amplitude and used to estimate the compressive strength of UHPC specimens. They found that the increase of compressive strength in UHPC leads to the decrease of both the integrated and the maximum SAR amplitudes. Kumar et al.^22^ used a 0.9GHz GPR to estimate the variability of in situ material properties (e.g. compressive strength) over time and space for the condition assessment of concrete bridges. Their results showed that attributes (or features) of the GPR data such as raw average amplitudes can be used to identify differences in compressive strength across the deck of a concrete bridge. Attributes such as instantaneous amplitudes and intensity of reflected GPR signals are useful in predicting material properties such as compressive strength, porosity, and density. Morris et al.^12^ studied the relationship between GPR (0.9GHz) image attributes and the mechanical properties (density, porosity, and compressive strength) of concrete by machine learning models. They found that there is a nonlinear relationship between the GPR image attributes and the compressive strength of concrete, which could be further characterized and predicted with a larger training set. Saleh et al.^23^ used RH with destructive core test using different model identification approaches to predict the compressive strength of concrete with small number of cores. Among the approaches they studied, they reported that the bi-objective approach is most efficient in estimating the mean compressive strength of concrete with the minimum number of cores. El-Mir et al.^24^ applied machine learning models to predict the compressive strength of concrete using RH, as a function of the w/c ratio (0.2 ~ 0.64) and concrete age (7, 28, and 56 days). They found that the integration of concrete age in the modeling can improve the compressive strength prediction of concrete with the rebound number for all w/c ratios and at all ages.

Combined uses of mechanical sensors and radar sensors for estimating the compressive strength of concrete have also been reported in the past. Qasrawi^10^ combined RH and UT on concrete cubes (6") of unknown history to correlate the rebound number and UPV for the compressive strength of concrete. From his result, it was found that the increase of compressive strength is associated with the increase of both rebound number and UPV. The rebound number and UPV was also found to be linearly correlated with a positive slope. Pucinotti^25^ used RH (type N) and UT with destructive core test to assess the in-situ compressive strength of concrete. He found that the combination of RH and UT can improve the coefficient of determination from 0.24 for RH and 0.82 for UT to 0.89 when they are combined. Ali-Benyahia et al.^26^ studied RH, UT (54 kHz), and the combination of both to estimate the compressive strength in real concrete structures. Statistical parameters such as probability distribution density function were developed from the rebound number and UPV of 205 concrete cores. From their analysis, the increase of compressive strength is associated with an approximately linear increase of the rebound number. But the increase of UPV was nonlinearly associated with the increase of the compressive strength. They also proposed a scaling calibrating method to improve the prediction accuracy for individual or combined use of RH and UT. Kouddane et al.^17^ investigated RH (type N) and UT (54 kHz) and the combination (SONicREBound or SonReb method) with the Monte Carlo method to predict the compressive strength of 205 concrete cores, using a multi-objective approach. In their experimental data, the rebound number showed a linear correlation with the compressive strength of concrete, while the UPV showed a nonlinear correlation with the compressive strength of concrete. Yuva^27^ combined RH and UT by using the SonReb method for estimating the compressive strength of concrete cubes. Bensaber et al.^28^ also applied the SonReb method to correlate RH and UT for estimating the flexural strength of concrete. From the result of our literature review, there was no research on the individual or the combined use of SAR for estimating the compressive strength of concrete.

The objective of this paper is to present a multiphysical NDE approach for the estimation of compressive strength of concrete using SAR, UT, and RH by considering four different w/c ratios (0.4, 0.45, 0.5, and 0.55) and at four different ages (7, 14, 28, and 96 days). Apart from the previous works, the novelty of this research is the use of SAR for multiphysical NDE. In what follows, our research approach (nondestructive SAR, UT, and RH / destructive compression test) is first provided, followed by experimental results and discussion. Empirical models based on individual and combined use of SAR, UT, and RH are proposed. Finally, our major findings are summarized in the conclusion.

## Research approach

### Design and manufacturing of concrete specimens

**Fig. 1:**
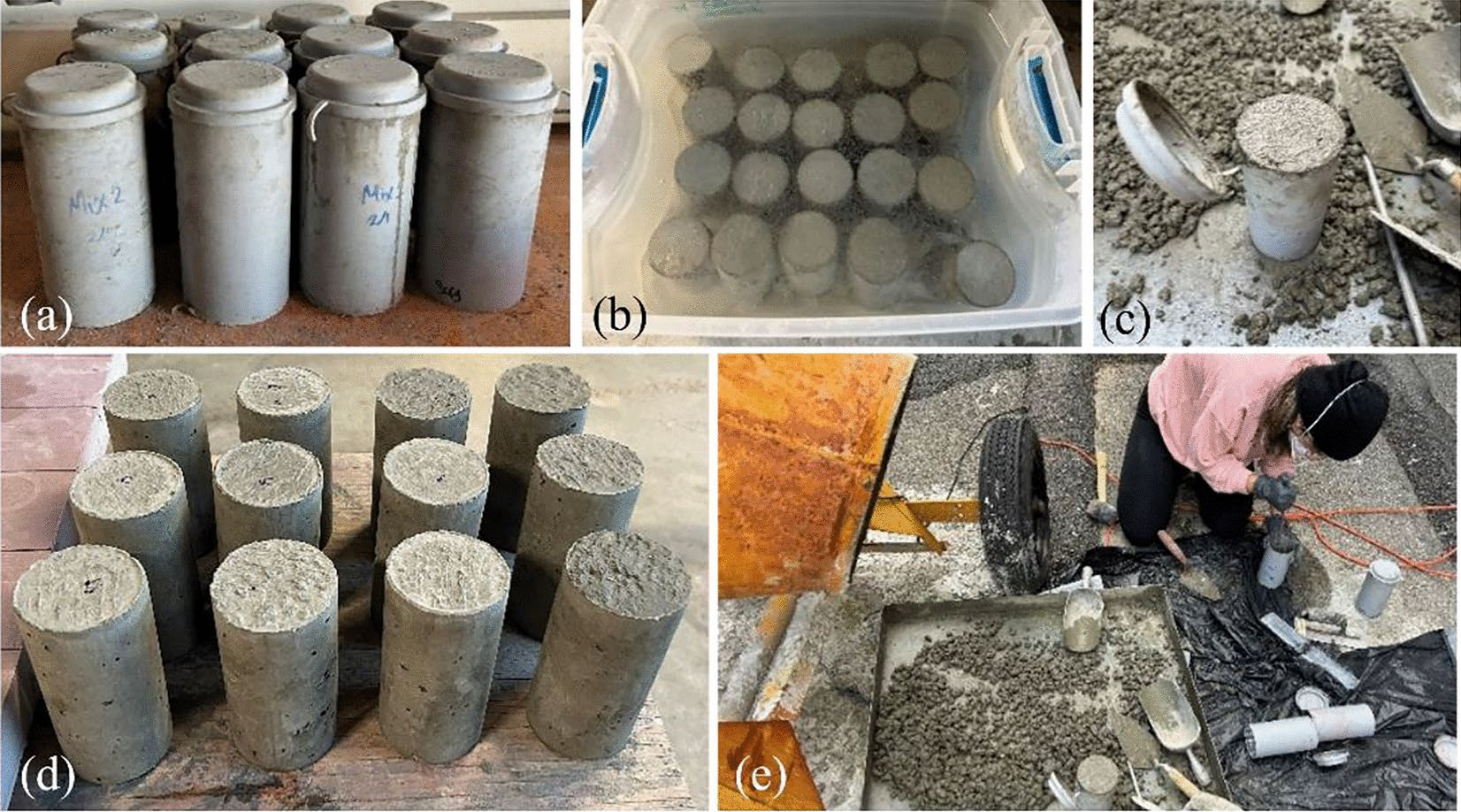
Photos of concrete specimens from laboratory casting and curing.

In this research, forty-eight concrete cylinder specimens (diameter = 3", height = 6") with a mix design ratio of 1:2:3 for cement, sand and gravel (by mass) and four different water-to-cement ratios (0.4, 0.45, 0.5, and 0.55) were manufactured and kept in a curing tank filled with lime (calcium hydroxide) water in room temperature. These specimens were created to obtain compressive strength of concrete in different ages (7, 14, 28, and 96 days) with three NDE methods (rebound hammer, UPV and SAR) in order find a correlation among three NDE methods. For every age and w/c ratio, three specimens were manufactured (A, B and C)^29^. For example, specimens C50A, C50B and C50C were named for a 0.5 w/c ratio and 7-day age. After one day of casting, the specimens were demolded and placed in a curing tank before NDE and destructive compression testing. Figure 1 shows the casting, compaction, demolding, and curing of concrete specimens. Table 1 lists the mix design of concrete used in this research.

**Table 1:** Concrete mix design.

w/c ratio	Cement (lb)	Sand (lb)	Gravel (lb)	Water (lb)
0.4	3.8760	7.7520	11.6258	1.6964
0.45	3.8012	7.6023	11.4080	1.8552
0.5	3.7286	7.4617	11.1948	2.0094
0.55	3.6633	7.3256	10.9907	2.1546

### Nondestructive evaluation techniques

#### Rebound hammer

**Fig. 2:**
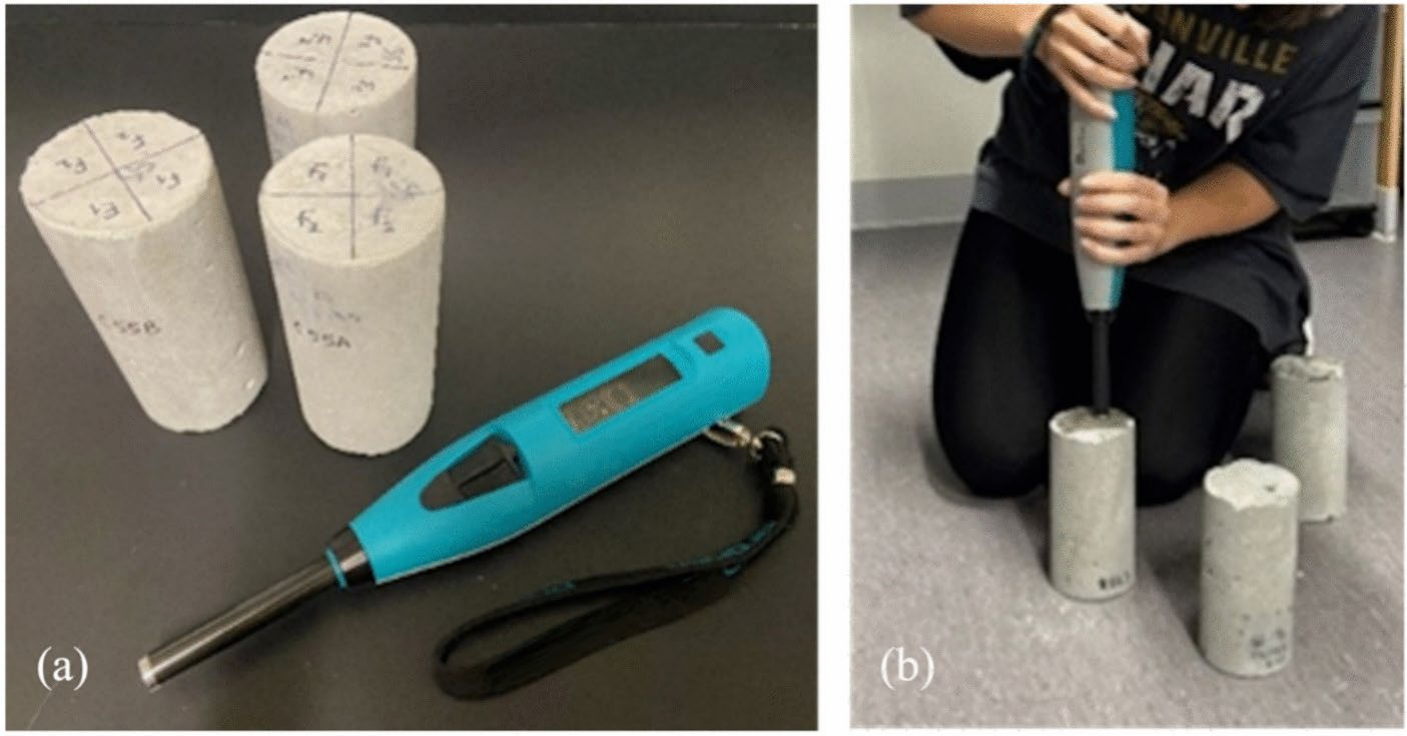
(**a**) rebound hammer and concrete cylinder specimens, (**b**) inspection scheme.

A commercially available rebound hammer (Schmidt Type N) was used as an NDE technique to estimate the compressive strength of concrete specimens. The rebound hammer consists of an outer body, a spring, a plunger, an indicator, a hammer mass, and a latch. When taking measurements, the rebound hammer is placed perpendicularly to the concrete surface to ensure the proper transmission of linear impulse into the concrete specimen under testing. Next, the rebound hammer is slowly pressed against the concrete surface to compress the spring (elastic potential energy) until the maximum compression is reached. Once the maximum compression of the spring is achieved, the latch will release the spring to move the hammer mass to impact the end of the outer body. The hammer mass will then be rebounded toward the concrete surface with an input linear impulse (or kinetic energy). The rebound linear impulse is finally measured by a rebound distance of the hammer mass. The rebound distance (or the Q value) is a numerical value between 10 and 100 and used to empirically indicate the compressive strength of concrete. According to ASTM C805^30^, ten readings at different impact points must be taken from each test area. A test area shall be at least 150 mm in diameter. The spatial distance between two impact points shall be at least 25 mm. The distance between all impact points and the edges of a concrete specimen or structure shall be at least 50 mm. During the data collection, all concrete specimens must be placed on a rigid surface to ensure that there is no relative movement between the rebound hammer and the concrete under testing. In principle, lower strength concretes will absorb more energy than higher strength concretes, resulting in a lower Q value. Figure 2 shows the rebound hammer used in this paper and the data collection scheme in the laboratory.

#### Ultrasonic testing

**Fig. 3:**
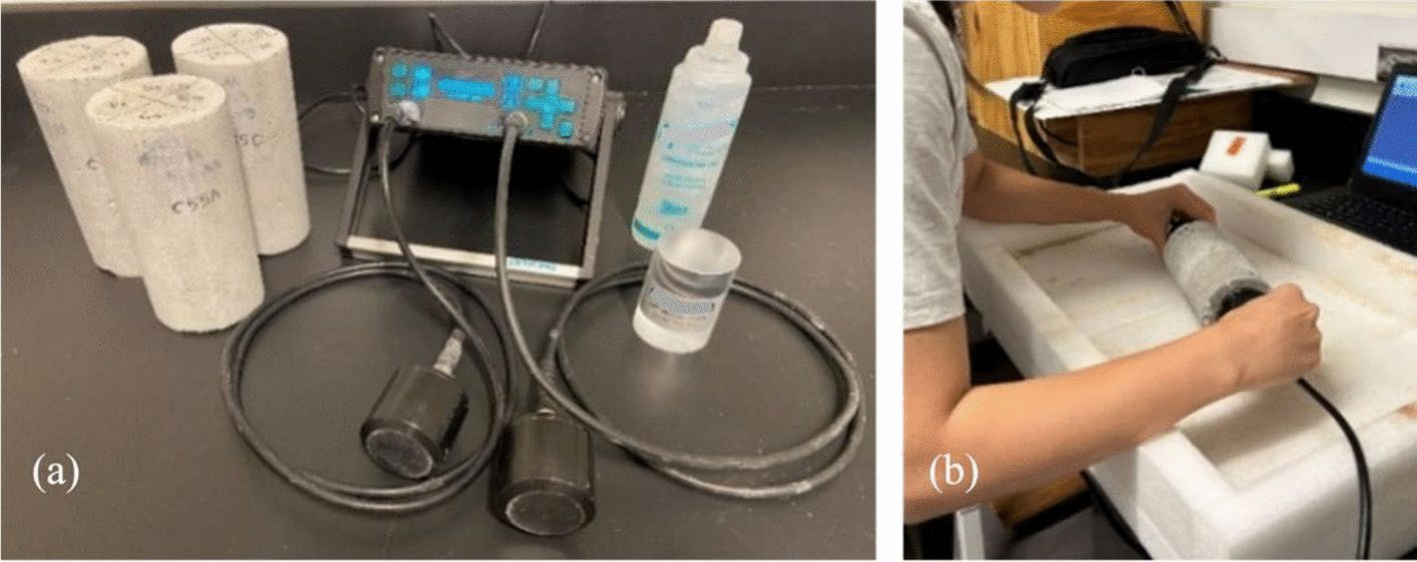
(**a**) Pundit Lab UT system; (**b**) measurement scheme.

In the ultrasonic testing (UT) of concrete compressive strength, we used two-port UT system (Pundit Lab, 54 kHz) to measure the ultrasonic pulse velocity (UPV) of concrete. This UT system is composed of a pulse generator, a pair of transducers (a transmitter and a receiver), a signal amplifier, and two connecting cables. The pulse generator produces repetitive short-duration pulses (in time domain) and sends them to the transmitter. The receiver measures the arrival time of these pulses. From the time difference between the transmitter and the receiver and a known travel distance, UPV can be calculated from concrete specimens. Among two basic modes (transmission and reflection) in UT, the transmission mode was used in our experimentation by placing the transmitter and the receiver at both ends of a concrete cylinder specimen. Figure 3 shows our UT system and measurement scheme. It is noteworthy to point out that care must be taken when conducting UT measurement on concrete specimens. Issues with surface coupling and alignment of transducers can significantly affect the quality and accuracy of measured UPV^31^. Equation (1) describes how UPV (or ${v}_{p}$) is related to material’s properties such as dynamic Young’s modulus *E* (Pa), Poisson’s ratio $\mu$, and density *ρ* (kg/m^3^).

1 $${v}_{p}=\sqrt{\frac{E(1-\mu )}{\rho (1+\mu )(1-2\mu )}}=\frac{L}{t},$$

where ${v}_{p}$ is the ultrasonic phase velocity of the P-waves (m/s), *L* the height of concrete cylinders (m), and *t* the traveling time of pulses (s).

#### SAR imaging

**Fig. 4:**
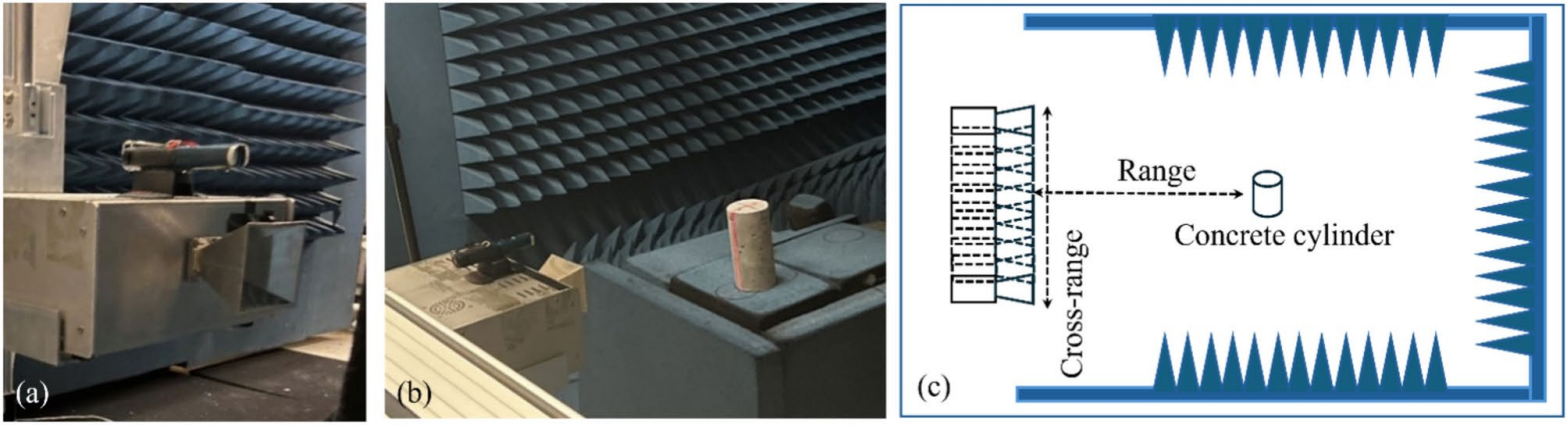
(**a**) SAR imaging radar; (**b**) concrete cylinder inside the chamber; (**c**) schematic of lab configuration.

In synthetic aperture radar (SAR) imaging, high-resolution coherent (continuous wave) images are produced with adjustable frequency bandwidth and artificial radar aperture. A laboratory monostatic (single antenna) SAR imaging system in Structural Engineering Research Group (SERG) in the Department of Civil and Environmental Engineering at UMass Lowell was used in this research. This SAR imaging system primarily consists of an imaging radar sensor, a 2D positioner, and an EM anechoic chamber (1 ~ 18 GHz) to perform stripmap SAR imaging of laboratory specimens. The radar sensor consists of a horn antenna, a signal generator, a signal modulator/demodulator, and a signal amplifier. The radar sensor travels at a spatial increment of 0.00625 m when generating SAR images of concrete specimens. The bandwidth and carrier frequency of the radar sensor are 1.5 GHz and 10 GHz, respectively. The use of this anechoic chamber enabled us to develop clean SAR images. Formulation of the SAR imaging algorithm used in this paper can be found in Ref.^18^. Figure 4 shows the photos and the schematic of SAR imaging inside the anechoic chamber.

Two SAR image parameters are defined and used in this research; the maximum SAR amplitude ${I}_{\text{max}}$ and the integrated SAR amplitude ${I}_{int}$.

2 $${I}_{\text{max}}=\underset{(r, {r}_{x})\in {\Omega }_{s}}{\text{max}}\left[I(r, {r}_{x})\right],$$

where $I(r, {r}_{x})$ is the SAR image in the range (*r*)-cross-range (*r*_*x*_) domain, *r* the range (in.) *r*_*x*_ the cross-range (in.), and ${\Omega }_{s}$ the scattering signal representing the concrete cylinder.

3 $${I}_{int}={\iint }_{\text{min}}^{\text{max}}I\left(r, {r}_{x}\right)drd{r}_{x},$$

where $\text{min}$ is the minimum values of $(r, {r}_{x})$ within the domain of ${\Omega }_{s}$, and $\text{max}$ the maximum values of $(r, {r}_{x})$ within the domain of ${\Omega }_{s}$.

### Destructive compression test

**Fig. 5:**
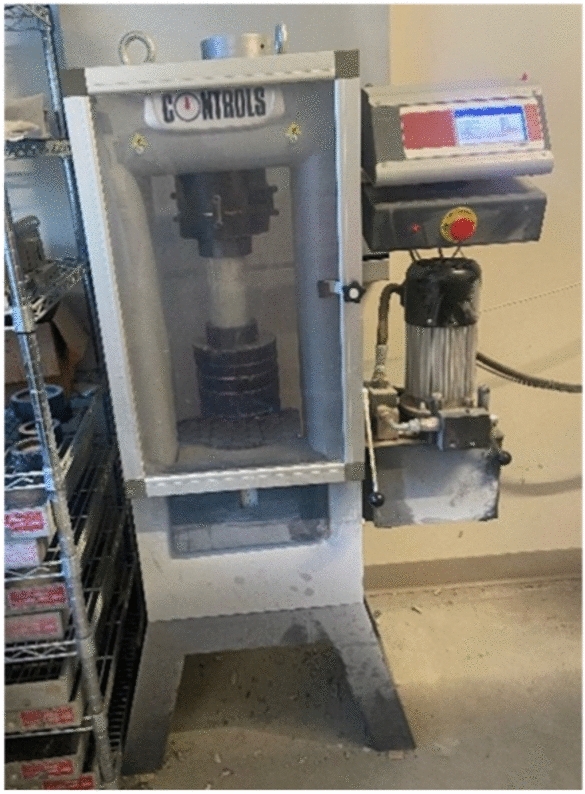
Compression testing machine.

A compression testing machine (Pilot Compact-Line Automatic Compression Machine, 200kN capacity) was used to measure the compressive strength of concrete specimens, which is comprised of a load frame, a load cell, a hydraulic system, and a data acquisition system. Compressive strength of concrete was determined by compressing concrete cubes or cylinders to failure. A time-depending loading curve was recorded during the test. Figure 5 shows the compression testing machine in the concrete laboratory.

The compressive strength ${f}_{c}$ of concrete cylinders was calculated by dividing the maximum load attained during the test by the cross-sectional area of a concrete cylinder. According to the ASTM C39^5^, three concrete cylinders were used when obtaining the average compression strength of concrete at different ages. Calculation of the concrete compressive strength is provided in Eq. (4).

4 $${f}_{c}=\frac{4{P}_{m}}{\pi {D}^{2}},$$

where ${f}_{c}$ is the concrete compressive strength (MPa), ${P}_{m}$ the measured maximum load (N), and *D* the diameter of concrete cylinders (mm).

## Results and discussion

### Destructive compression test

**Fig. 6:**
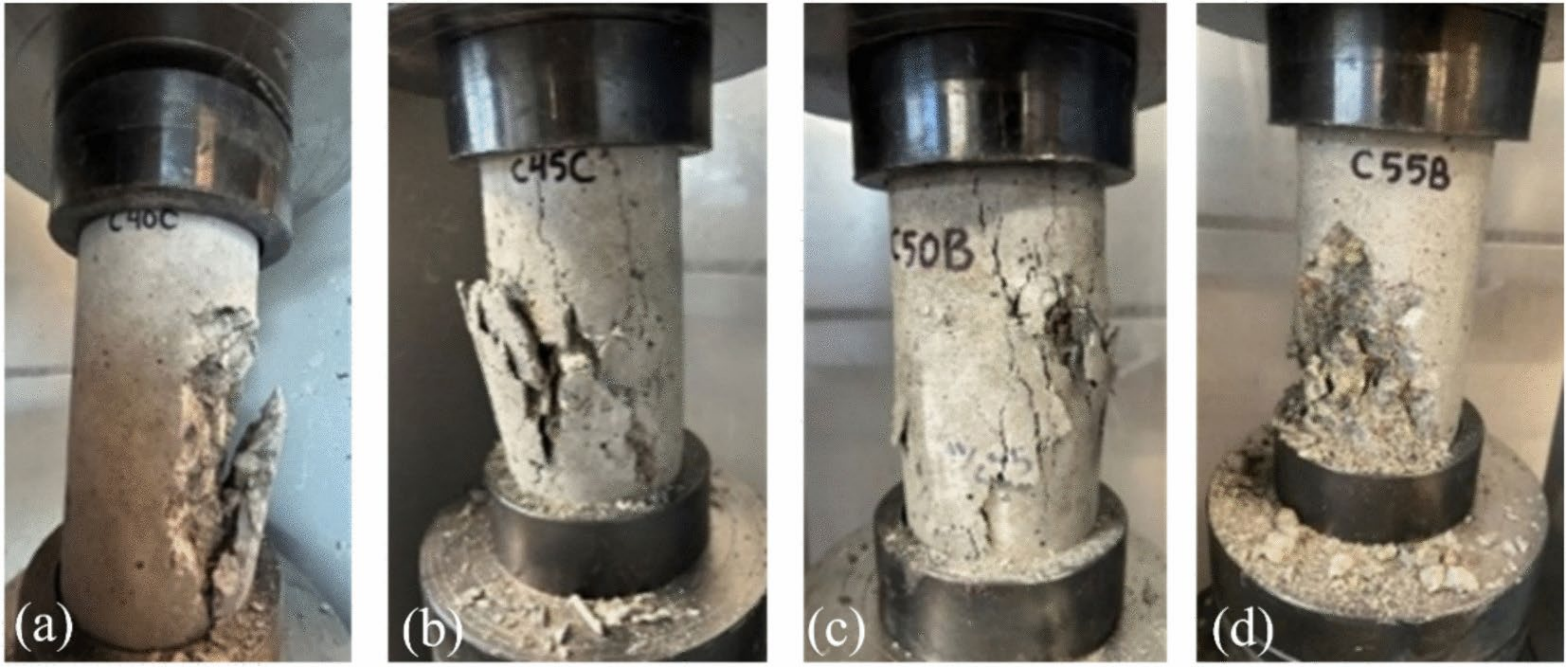
Failure modes of concrete cylinders; (**a**) vertical cracking (**b**) vertical cracking, (**c**) vertical cracking, and (**d**) split and shear.

After conducting the compression test of concrete cylinders on four different dates, the compressive strength of concrete cylinders was obtained as a function of time. Almost all concrete cylinders failed in vertical cracking or combined split and shear. No concrete cylinders failed in local failure modes (e.g., chipped corner). Figure 6 shows typical failures of concrete cylinders in this paper. Table 2 lists the measured compressive strength of concrete cylinders at four different ages.

**Table 2:** Measured compressive strength (in MPa) of concrete cylinders.

w/c ratio	Time			
	7 days	14 days	28 days	96 days
0.4	38.4741	40.7307	45.6591	49.0279
0.45	32.8997	33.1989	41.2437	41.3237
0.5	26.7557	32.9459	36.1181	38.9664
0.55	25.2534	26.7164	29.6757	35.6872

**Fig. 7:**
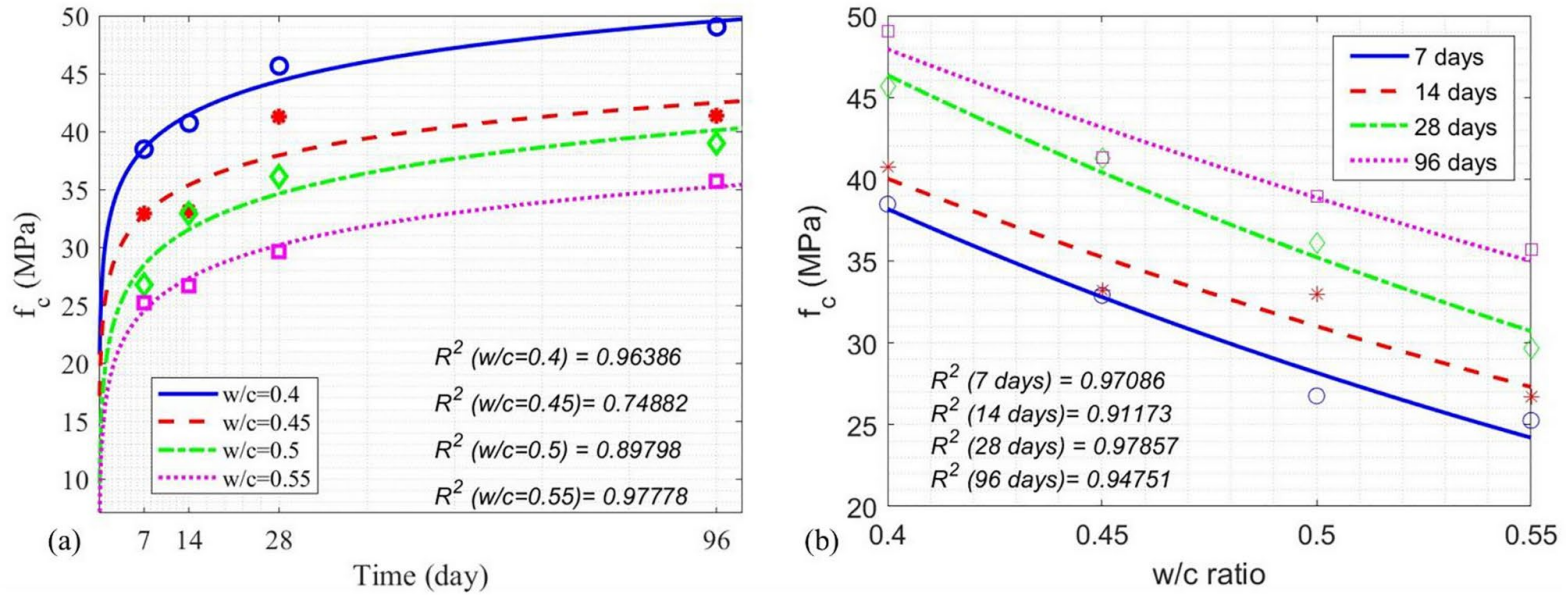
(**a**) Measured concrete compressive strength and model curves as a function of time, (**b**) measured concrete compressive strength and model curves as a function of the w/c ratio.

Measured concrete compressive strength data on four different dates (7, 14, 28, and 96 days) with four w/c ratios (0.4, 0.45, 0.5, and 0.55) are shown in Fig. 7. In Fig. 7a, it is clear that the concrete compressive strength increases with the age of concrete. In Fig. 7b, it is also expected that the increase of the w/c ratio leads to the decrease of concrete compressive strength. Equations (3) to (6) were empirically developed from our experimental data in Fig. 7a.

5 $${\left.{f}_{c}\left(t\right)\right|}_{z=0.4}=2.904{\text{log}}_{2}t+30.398,$$

6 $${\left.{f}_{c}\left(t\right)\right|}_{z=0.45}=2.554{\text{log}}_{2}t+25.691,$$

7 $${\left.{f}_{c}\left(t\right)\right|}_{z=0.5}=3.081{\text{log}}_{2}t+19.822,$$

8 $${\left.{f}_{c}\left(t\right)\right|}_{z=0.55}=2.835{\text{log}}_{2}t+16.57,$$

where *z* is the w/c ratio and *t* the concrete age (in days). Equations (7) to (10) were empirically developed from our experimental data in Fig. 7b.

9 $${\left.{f}_{c}\left(z\right)\right|}_{t=7d}=128.86 {e}^{(-3.04z)},$$

10 $${\left.{f}_{c}\left(z\right)\right|}_{t=14d}=111.074{e}^{(-2.552z)},$$

11 $${\left.{f}_{c}\left(z\right)\right|}_{t=28d}=138.998 {e}^{(-2.745z)},$$

12 $${\left.{f}_{c}\left(z\right)\right|}_{t=96d}=110.94 {e}^{(-2.098z)}.$$

Performance of these empirical models relating the w/c ratio and concrete age to the concrete compressive strength is also shown in Fig. 7a and b. These results demonstrate good quality control in the manufacturing of our concrete specimens.

### Rebound hammer for compressive strength prediction

Since our specimens were smaller than 150 mm (diameter = 3" or 76.2 mm), the average of six points on the surface area was calculated as the measured Q value. Table 3 lists all measured Q values of all concrete cylinders at four different ages.

**Table 3:** Measured Q values of concrete cylinders.

w/c ratio	Time			
	7 days	14 days	28 days	96 days
0.4	36.23	36.5	37.5	38
0.45	34.3	34.7	36.53	36.5
0.5	32.8	34.2	35	35.8
0.55	32.06	32.5	33.5	35

**Fig. 8:**
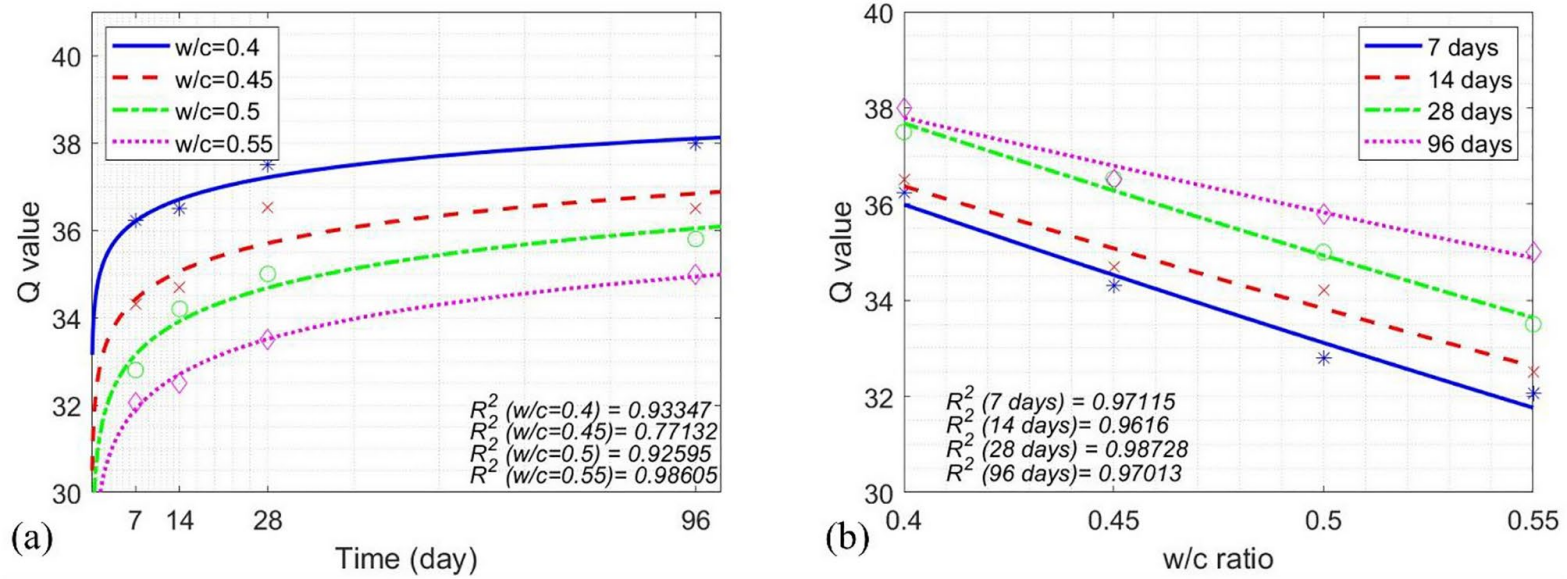
(**a**) Measured Q-values of concrete cylinders and model curves as a function of time, (**b**) measured Q-values of concrete cylinders and model curves as a function of the w/c ratio.

An empirical model was developed to model the time-dependent Q values for each w/c ratio at four different ages, as shown in Eq. (4). Model performance is illustrated in Fig. 8a as a function of time and Fig. 8b as a function of the w/c ratio (denoted by *z*).

13 $${\left.Q\left(t\right)\right|}_{z=0.4}=0.4993{\text{log}}_{2}t+34.81,$$

14 $${\left.Q\left(t\right)\right|}_{z=0.45}=0.6405{\text{log}}_{2}t+32.62,$$

15 $${\left.Q\left(t\right)\right|}_{z=0.5}=0.7641{\text{log}}_{2}t+31.01,$$

16 $${\left.Q\left(t\right)\right|}_{z=0.55}=0.8039{\text{log}}_{2}t+29.65,$$

17 $${\left.Q\left(z\right)\right|}_{t=7d}=50.23{e}^{(-0.8335z)},$$

18 $${\left.Q\left(z\right)\right|}_{t=14d}=48.62{e}^{(-0.7255z)},$$

19 $${\left.Q\left(z\right)\right|}_{t=28d}=51.01{e}^{(-0.757z)},$$

20 $${\left.Q\left(z\right)\right|}_{t=96d}=46.84{e}^{(-0.5361z)}.$$

The average R^2^ value was 0.89711 for the time-dependent *Q* models in Eqs. (13) ~ (16), as shown in Fig. 8a, and the average R^2^ value was 0.95217 for the w/c ratio-dependent *Q* models Eqs. (17) ~ (20), as shown in Fig. 8b. In Fig. 8, it is clear that the increase of concrete age leads to the nonlinear increase of measured *Q* values of concrete. A lower w/c ratio resulted in a greater measured Q value. The increase of *Q* values was in good agreement with the growth of concrete compressive strength, as expected.

**Fig. 9:**
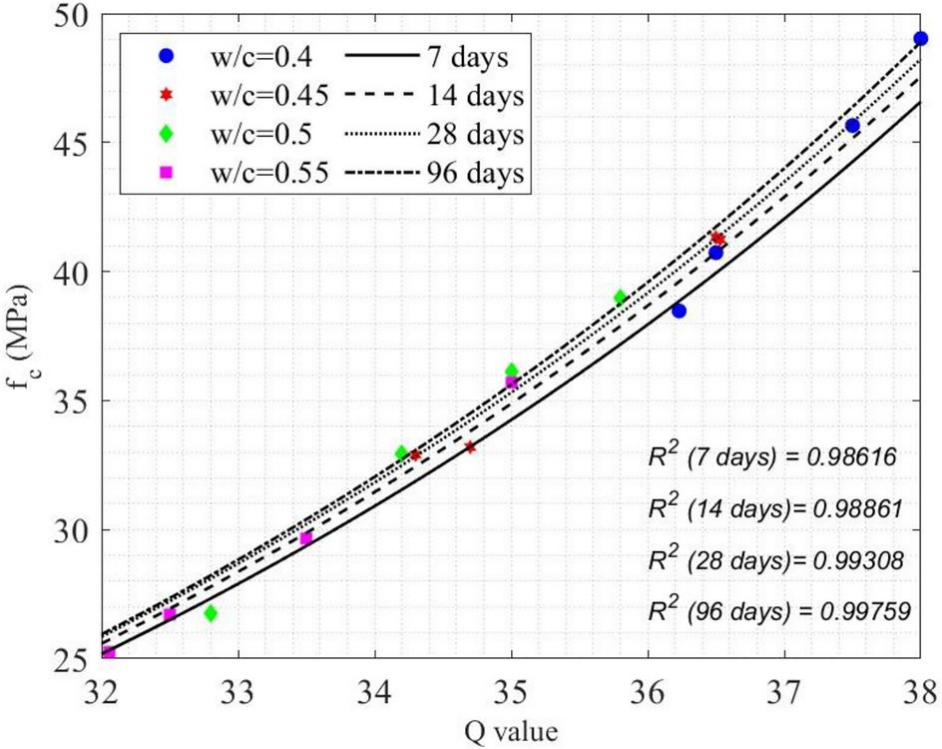
Experimental data and the models between Q-value and compressive strength of concrete.

To predict the compressive strength of concrete using measured *Q* values, concrete age *t*, and the w/c ratio (*z*), the following model was proposed in Eq. (21).

21 $${f}_{c}\left(\text{Q},z, t\right)=\left(-6.964\times {10}^{-4}tz+0.9491\right){e}^{0.1027Q},$$

where *t* is the concrete age ($t\in \left[7, 96\right]$, in days), Q $\in \left[32.06, 38\right]$, and z the w/c ratio (*z* $\in \left[0.4, 0.55\right]$). This model delivered an average R^2^ value of 0.9914 as a function of the *Q* value, whose performance is illustrated in Fig. 9. Table 4 lists the percentage error between the model prediction and the measured ${f}_{c}$.

**Table 4:** Percentage error of Eq. (19) on compressive strength prediction.

w/c ratio	Time			
	7 days	14 days	28 days	96 days
0.4	1.6724	1.4631	2.9938	6.8132
0.45	2.5040	0.4350	2.8920	5.5676
0.5	2.7398	3.9083	5.3348	7.1407
0.55	0.8603	0.5376	1.3352	6.9477

### UPV for compressive strength prediction

Table 5 lists all measured UPV (${v}_{p}$) values of concrete cylinders of four w/c ratios at four different ages. From the measured UPV values, ${v}_{p}$ increased with the increase of concrete age, as expected. Four empirical models.

**Table 5:** Measured ${v}_{p}$(m/s) of concrete cylinders.

w/c ratio	Time			
	7 days	14 days	28 days	96 days
0.4	5036	5048.7	5097.58	5112
0.45	4951.6	4957	5050	5057
0.5	4864.3	4917	4953.6	4990
0.55	4811.25	4836	4870.1	4940

The time-dependence of ${v}_{p}$ was modeled for four w/c ratios in Eqs. (22) ~ (25), and the w/c ratio-dependence of ${v}_{p}$ was modeled for four concrete ages in Eqs. (26) ~ (29).

22 $${\left.{v}_{p}\left(t\right)\right|}_{z=0.4}=31.19{\text{log}}_{2}t+4976,$$

23 $${\left.{v}_{p}\left(t\right)\right|}_{z=0.45}=45.56{\text{log}}_{2}t+4862,$$

24 $${\left.{v}_{p}\left(t\right)\right|}_{z=0.5}=46.79{\text{log}}_{2}t+4785,$$

25 $${\left.{v}_{p}\left(t\right)\right|}_{z=0.55}=61.42{\text{log}}_{2}t+4680,$$

26 $${\left.{v}_{p}\left(z\right)\right|}_{t=7d}=5695{e}^{(-0.3102z)},$$

27 $${\left.{v}_{p}\left(z\right)\right|}_{t=14d}=5627{e}^{(-0.2747z)},$$

28 $${\left.{v}_{p}\left(z\right)\right|}_{t=28d}=5788{e}^{(-0.3115z)},$$

29 $${\left.{v}_{p}\left(z\right)\right|}_{t=96d}=5610{e}^{(-0.2321z)}.$$

The average R^2^ value was 0.8982 for the time-dependent ${v}_{p}$ models in Eqs. (22) ~ (25) and 0.9876 for the w/c ratio-dependent ${v}_{p}$ models in Eqs. (26) ~ (29).

**Fig. 10:**
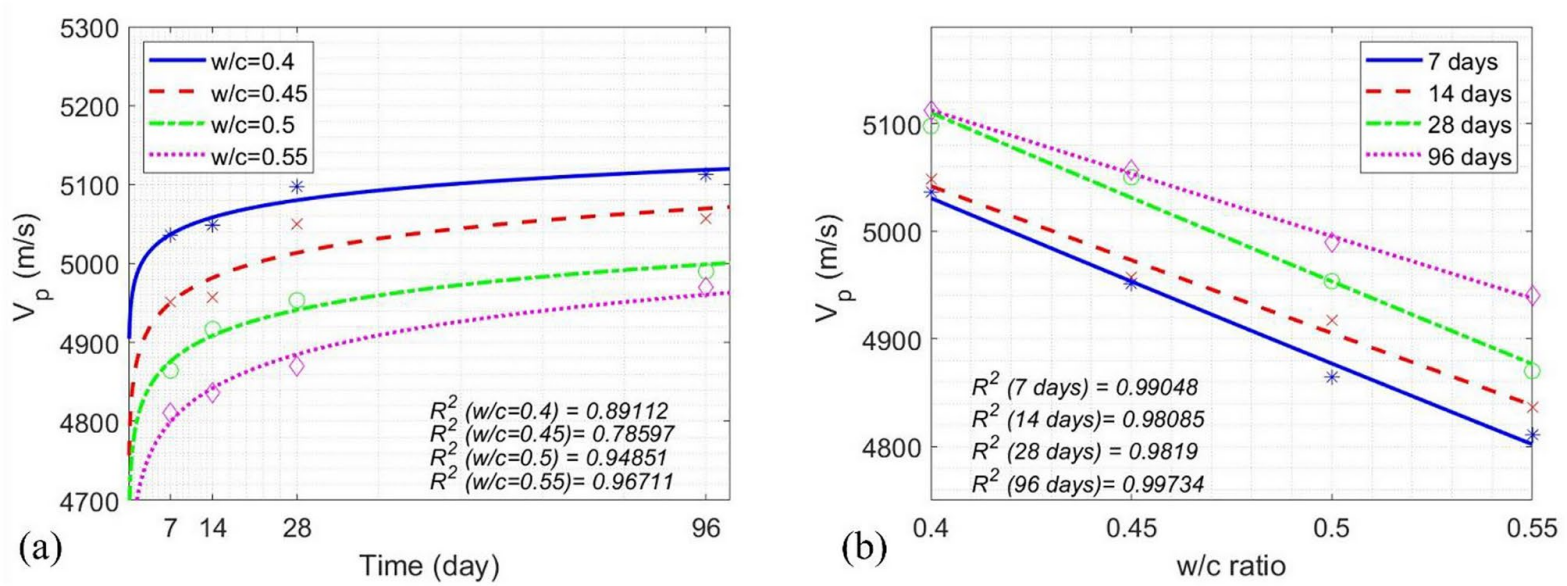
(**a**) Average ${v}_{p}$ of concrete cylinders vs. concrete age; (**b**) Average ${v}_{p}$ of concrete cylinders vs. the w/c ratio.

**Fig. 11:**
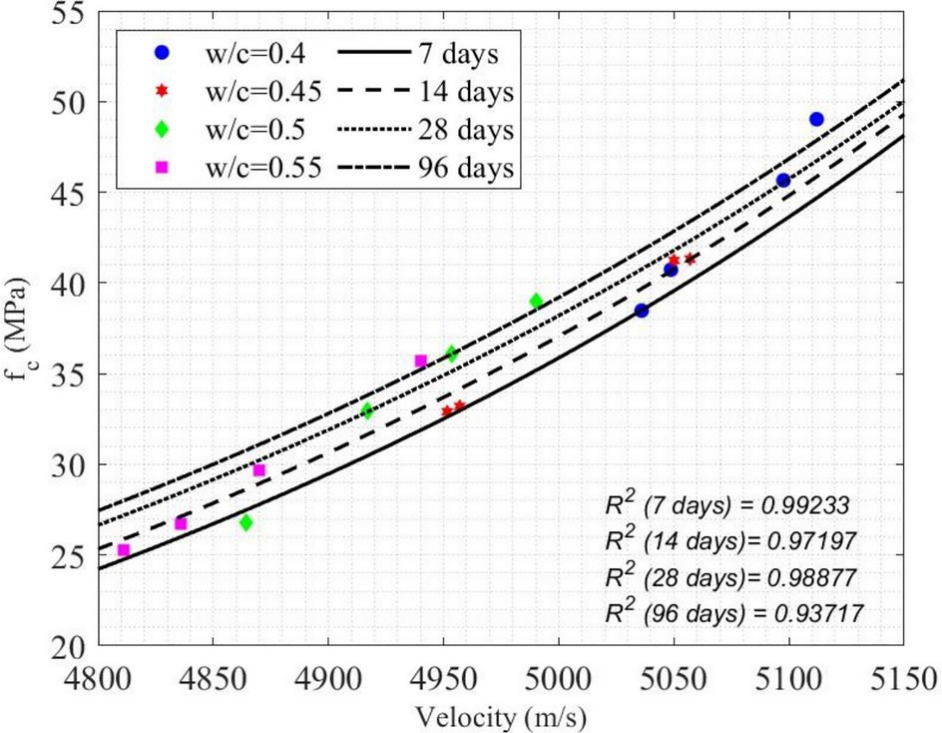
Experimental data and the models between UPV and the compressive strength of concrete.

Since ${v}_{p}$ and the Q value are all fundamentally related to the mechanical property of concrete, ${v}_{p}$ curves demonstrated a similar trend as the one of the Q value curves. Figure 10 illustrates the increase of ${v}_{p}$ over time for all four w/c ratios at four different ages. To predict the compressive strength of concrete using measured ${v}_{p}$ values, concrete age *t*, and the w/c ratio (*z*), the following model was proposed in Eq. (30).

30 $${f}_{c}\left({v}_{p},z, t\right)=\left(3.1716\times {10}^{-5}{t}^{z}+0.0053\right){e}^{0.0019{v}_{p}},$$

where *t* is the concrete age ($t\in \left[7, 96\right]$, in days), ${v}_{p}$ the ultrasonic pulse velocity (${v}_{p}\in \left[\text{4,811.25}, \text{5,112}\right]$, in m/s), and z the w/c ratio (*z* $\in \left[0.4, 0.55\right]$). Equation (30) represents the summary result for concrete compressive strength prediction using UPV, while Eq. (21) represents the summary result for concrete compressive strength prediction using the Q value (Fig. 11, Table 6),

**Table 6:** Percentage error of Eq. (30) on compressive strength prediction.

w/c ratio	Time			
	7 days	14 days	28 days	96 days
0.4	0.337	5.7	6.665	8.082
0.45	0.348	2.33	4.828	0.0573
0.5	0.085	8.274	8.64	4.443
0.55	0.308	2.404	4.05	2.462

### Conversion between ${\text{v}}_{\text{p}}$ and the Q value

Since both the Q value and UPV are based on the mechanical properties (Young’s modulus and Poisson’s ratio) of concrete, it is not surprising to see that the Q value and UPV of concrete share a similar correlation with *f*_*c*_. It is also well-known that the increase of *f*_*c*_ (due to cement hydration) is attributed to the increase of Young’s modulus and the decrease of Poisson’s ratio. With Eqs. (31) and (32), two conversion equations were developed from our data sets.

31 $${v}_{p}\left(Q, t, z\right)=54.053Q+\text{ln}\left[\sqrt[0.0019]{\frac{137.6615-0.101tz}{0.0046{t}^{z}+0.3669}}\right],$$

32 $$Q\left({v}_{p}, t, z\right)=0.0185{v}_{p}+\text{ln}\left[\sqrt[0.1027]{\frac{137.6615-0.101tz}{0.0046{t}^{z}+0.3669}}\right],$$

From our analysis and in the range of considered model parameters, it is found that the ultrasonic testing (transmissive mechanical sensing) delivered a superior performance over rebound hammer (reflective mechanical sensing) on the compressive strength prediction of concrete.

### SAR images for compressive strength prediction

SAR images for concrete cylinders at four different ages (days) with four different w/c ratios were generated in Fig. 13. To quantify these SAR images, the use of the ratio ${I}_{\text{SAR}}$ between the integrated SAR amplitude ${I}_{int}$ and the maximum SAR amplitude ${I}_{\text{max}}$ was proposed, which is

33 $${I}_{\text{SAR}}=\frac{ {I}_{int}}{{I}_{\text{max}}}.$$

**Fig. 12:**
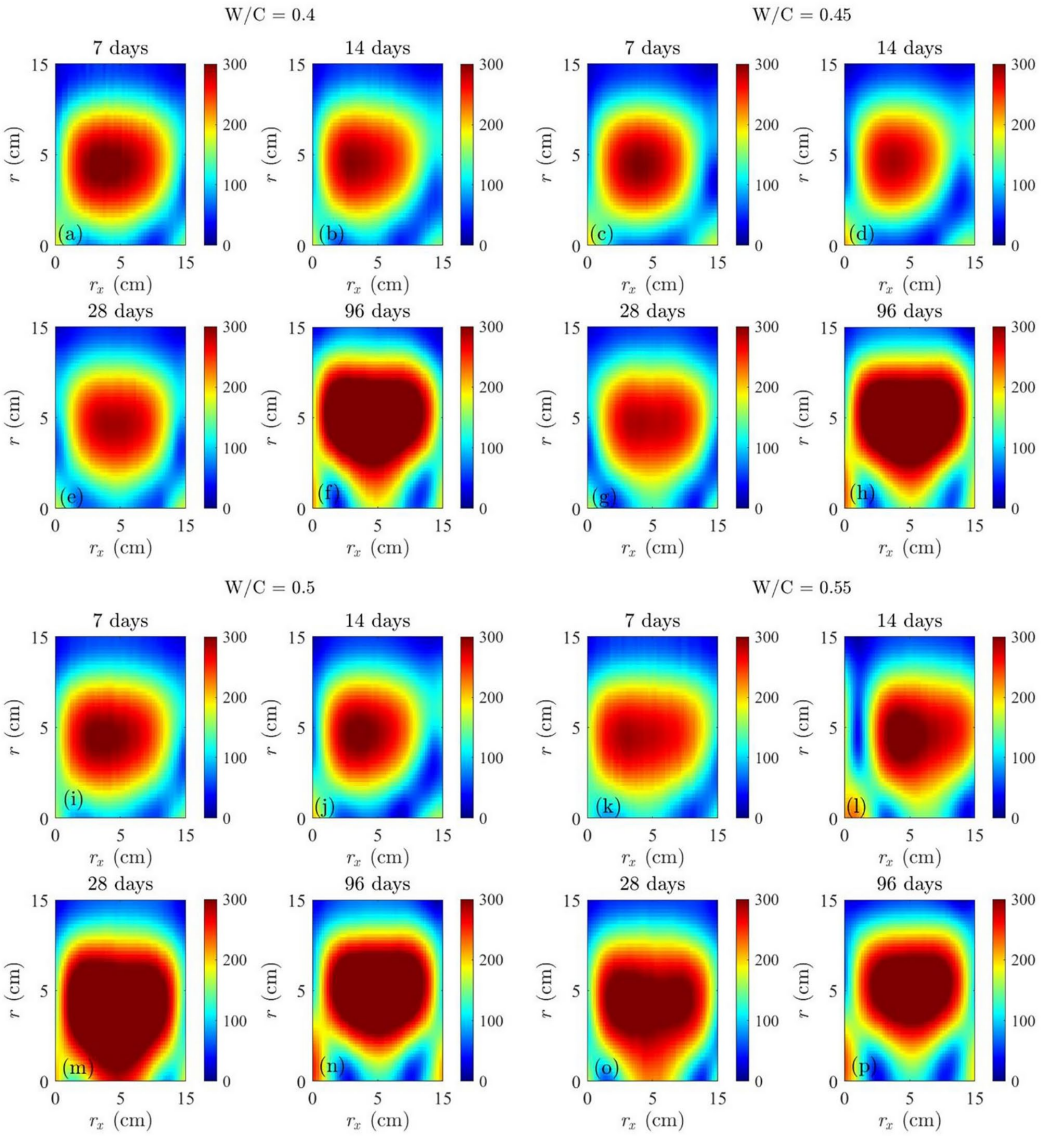
SAR images of concrete cylinders at different ages with different w/c ratios.

Figure 12 shows the performance of ${I}_{\text{SAR}}$ for all concrete cylinders.

**Fig. 13:**
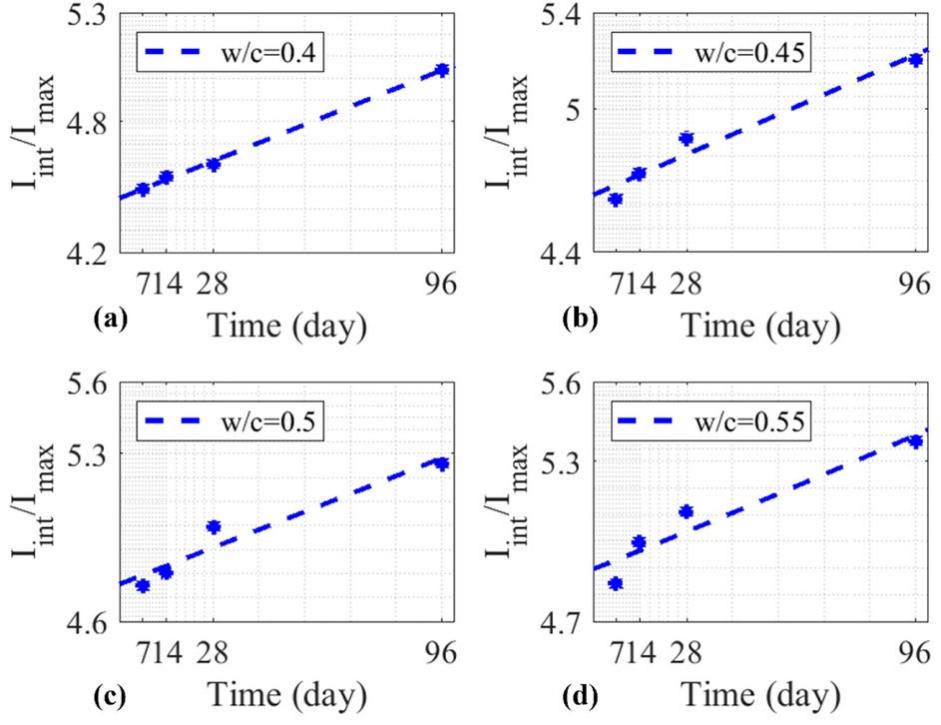
${I}_{int} /{I}_{max}$ of concrete cylinders at different ages with different w/c ratios.

**Fig. 14:**
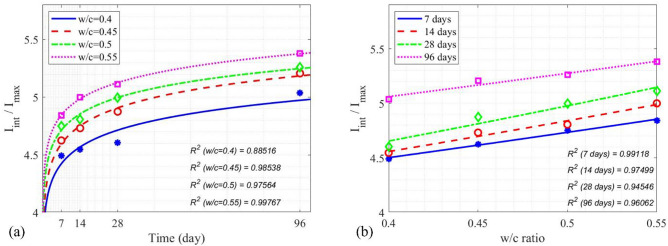
(**a**) ${I}_{int}/{I}_{max}$ of SAR images vs. concrete age, (**b**) ${I}_{int}/{I}_{max}$ of SAR images vs. the w/c ratio.

Concrete cylinders with higher w/c ratios are expected to have a higher dielectric constant due to a higher pore water content, as seen in the increase of ${I}_{int}/{I}_{max}$ in Fig. 13. Table 7 lists the calculated ${I}_{int}/{I}_{max}$ for all concrete cylinders. Figure 14 illustrates ${I}_{int}/{I}_{max}$ as a function of time and the w/c ratio, in which the trend of an increasing ${I}_{int}/{I}_{max}$ is associated with the increase of both concrete age and the w/c ratio.

**Table 7:** Measured $\frac{{{\varvec{I}}}_{{\varvec{i}}{\varvec{n}}{\varvec{t}}}}{{{\varvec{I}}}_{{\varvec{m}}{\varvec{a}}{\varvec{x}}}}$ for the prediction of compressive strength.

w/c ratio	Time			
	7 days	14 days	28 days	96 days
0.4	4.4808	4.5462	4.6037	5.0374
0.45	4.6245	4.7314	4.8748	5.2072
0.5	4.7495	4.8058	4.9963	5.2616
0.55	4.8409	4.9991	5.1139	5.3797

34 $${\left.{I}_{int}/{I}_{max}\left(t\right)\right|}_{z=0.4}=0.1457{\text{log}}_{2}t+4.013,$$

35 $${\left.{I}_{int}/{I}_{max}\left(t\right)\right|}_{z=0.45}=0.1562{\text{log}}_{2}t+4.156,$$

36 $${\left.{I}_{int}/{I}_{max}\left(t\right)\right|}_{z=0.5}=0.1417{\text{log}}_{2}t+4.316,$$

37 $${\left.{I}_{int}/{I}_{max}\left(t\right)\right|}_{z=0.55}=0.1408{\text{log}}_{2}t+4.45,$$

38 $${\left.{I}_{int}/{I}_{max}\left(z\right)\right|}_{t=7d}=3.683{e}^{(0.5017z)},$$

39 $${\left.{I}_{int}/{I}_{max}\left(z\right)\right|}_{t=14d}=3.584{e}^{(0.6009z)},$$

40 $${\left.{I}_{int}/{I}_{max}\left(z\right)\right|}_{t=28d}=3.559{e}^{(0.6706z)},$$

41 $${\left.{I}_{int}/{I}_{max}\left(z\right)\right|}_{t=96d}=4.29{e}^{(0.4133z)}.$$

**Fig. 15:**
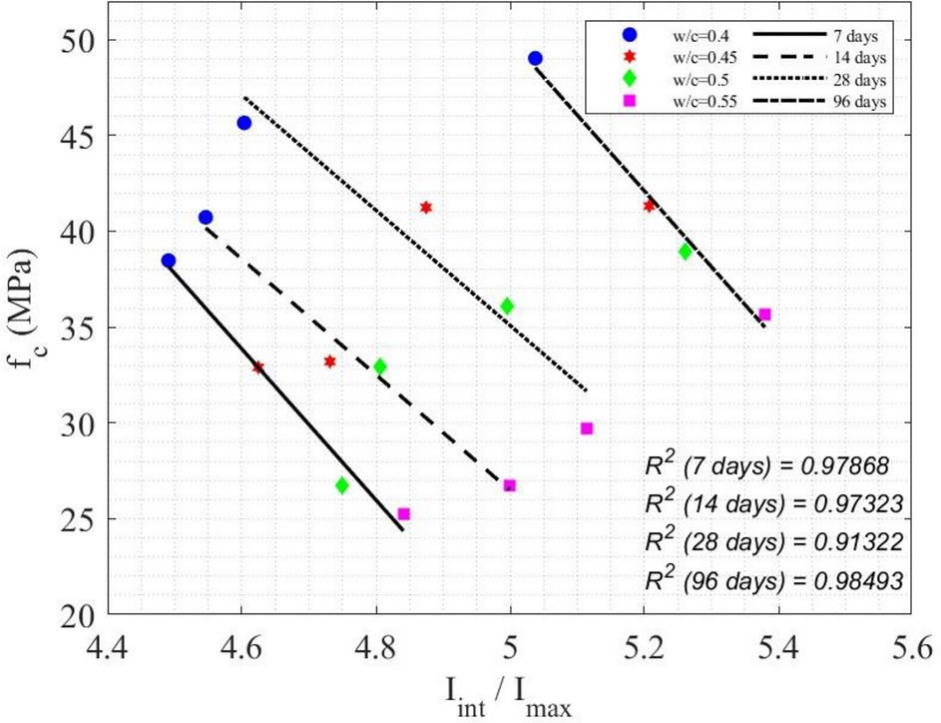
Experimental data and models between ${f}_{c}$ and ${I}_{int}/{I}_{max}$.

In Fig. 15, the relation between the normalized integrated SAR amplitude ${I}_{int}/{I}_{max}$ and ${f}_{c}$ of concrete cylinders is shown. It is evident that as the curing time increased, both the compressive strength ${f}_{c}$ and ${I}_{int}/{I}_{max}$ increased. Equivalently, with an increase in the w/c ratio, the compressive strength ${f}_{c}$ decreases, while ${I}_{int}/{I}_{max}$ increases. An empirical model for SAR image parameter to predict the compressive strength of concrete was proposed, as a function of age (t), water-to-cement ratio (z) and normalized integrated amplitude (${I}_{int}/{I}_{max}$) in Eqs. (42) and (43). Table 8 illustrates the measured error between the predicted ${f}_{c}$ values obtained from Eqs. (42) and (43), and the ${f}_{c}$ values from compression testing machines at four different ages for four w/c ratios. Figure 15 depicts the performance of Eqs. (42) and (43), with a R^2^ value ranging from 0.91322 to 0.98493, indicating a satisfactory performance of the models.

42 $${f}_{c}\left({I}_{SAR},z, t\right)=\left(-39.4035-0.00198t\right){I}_{SAR}+0.3686\left(t-z\right)+212.5792,$$

where ${I}_{SAR}={I}_{int}/{I}_{max}$ and t is the concrete age on 7 days and 96 days.

43 $${f}_{c}\left({I}_{SAR},z, t\right)=\left(-30.3369-0.0101t\right){I}_{SAR}+0.565\left(t-z\right)+169.5421,$$

where *t* is the concrete age on 14 days and 28 days.

**Table 8:** Percentage error of Eqs. (42) and (43) for the prediction of compressive strength.

w/c	Time			
	7 days	14 days	28 days	96 days
0.4	1.2361	1.9224	2.4209	1.3371
0.45	0.5772	3.3953	6.4286	0.7468
0.5	3.7666	2.7161	3.3461	1.2587
0.55	4.4020	1.9759	5.6296	2.5881

### Combined use of NDE techniques for compressive strength prediction

**Fig. 16:**
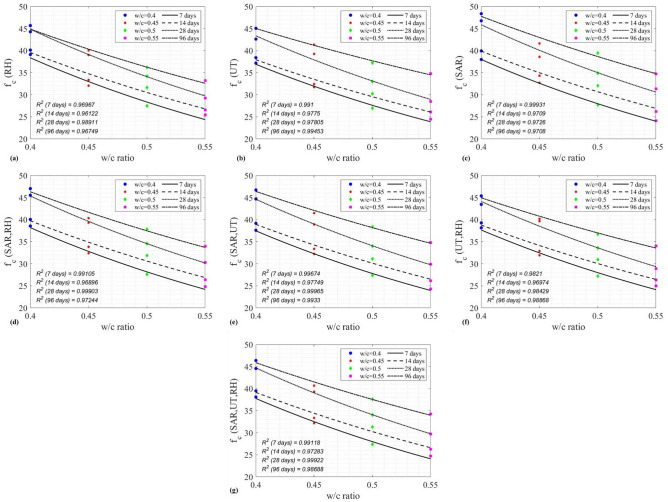
Model performance of (**a**) ${f}_{c}(Q)$, (**b**) ${f}_{c}({v}_{p})$, (**c**) ${f}_{c}\left({I}_{SAR}\right)$, (**d**) ${f}_{c}\left({I}_{SAR},Q\right)$, (**e**) ${f}_{c}\left({I}_{SAR},{v}_{p}\right)$, (**f**) ${f}_{c}\left({v}_{p},Q\right)$, (**g**) ${f}_{c}\left({I}_{SAR},Q,{v}_{p}\right)$.

**Fig. 17:**
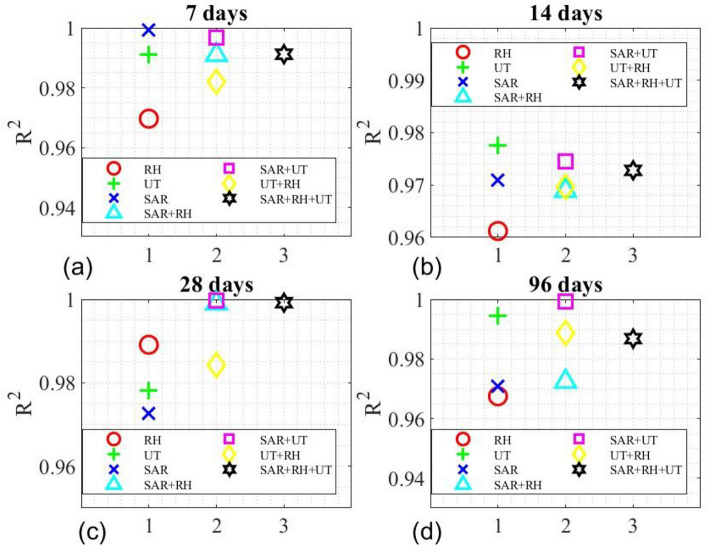
Comparison of ${R}^{2}$ values for individual and combined NDE techniques at different ages.

**Fig. 18:**
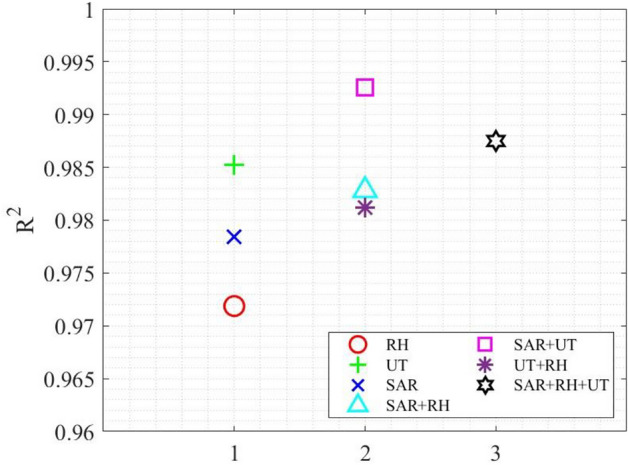
Overall comparison of individual and combined NDE techniques by ${R}^{2}$.

Combined use of three NDE techniques for compressive strength prediction of concrete was also investigated. In this analysis, a uniform-weighting approach was adopted, as shown in Eqs. (44) to (47). For a given combination (*t, z*) of concrete age and the water-to-cement ratio, its compressive strength can be estimated by

44 $${f}_{c}\left({I}_{SAR},Q\right)=\frac{1}{2}\left[{f}_{c}\left({I}_{SAR}\right)+{f}_{c}(Q)\right],$$

45 $${f}_{c}\left({I}_{SAR},{v}_{p}\right)=\frac{1}{2}\left[{f}_{c}\left({I}_{SAR}\right)+{f}_{c}({v}_{p})\right],$$

46 $${f}_{c}\left({v}_{p},Q\right)=\frac{1}{2}\left[{f}_{c}\left({v}_{p}\right)+{f}_{c}(Q)\right],$$

47 $${f}_{c}\left({I}_{SAR},Q,{v}_{p}\right)=\frac{1}{3}\left[{f}_{c}\left({I}_{SAR}\right)+{f}_{c}\left({I}_{SAR}\right)+{f}_{c}({v}_{p})\right],$$

where ${f}_{c}\left({I}_{SAR}\right)$ is either Eq. (42) or (43). ${f}_{c}(Q)$ is Eq. (21). ${f}_{c}\left({v}_{p}\right)$ is Eq. (30). Predicted compressive strength by individual NDE techniques is illustrated in Fig. 16a–c. Predicted compressive strength by various combined NDE techniques is shown in Fig. 16d–g. Performance of individual and combined NDE techniques is evaluated and compared in Table 9 by the coefficient of determination (R^2^) between model predictions and experimental measurements. Figure 17 summarizes all models on four different days, while Fig. 18 compares the performance of individual and combined NDE techniques. In Fig. 18, it is interesting to point out that, when combining two NDE techniques, the best combination is SAR with UT, followed by SAR with RH and UT with RH. It is also noteworthy to point out that the use of SAR improves the performance of either UT or RH, demonstrating the promising potential of SAR in multiphysical NDE.

**Table 9:** Performance of combined NDE techniques for ${f}_{c}$ by R^2^.

Combination	Time				
	7 days	14 days	28 days	96 days	Average
${f}_{c}(RH)$	0.96967	0.96122	0.98911	0.96749	0.97187
${f}_{c}(UT)$	0.991	0.9775	0.97805	0.99453	0.98527
${f}_{c}(SAR)$	0.99931	0.9709	0.9726	0.9708	0.9784
${f}_{c}(SAR+RH)$	0.99105	0.96896	0.99903	0.97244	0.98287
${f}_{c}(SAR+UT)$	0.99674	0.97749	0.99965	0.9933	0.9918
${f}_{c}(UT+RH)$	0.9821	0.96974	0.98429	0.98868	0.9812
${f}_{c}(SAR+RH+UT)$	0.99118	0.97283	0.99922	0.9868	0.98751

## Conclusion

In this paper, our experimental and modeling efforts on the compressive strength prediction of concrete cylinders with four different w/c ratios (0.4, 0.45, 0.5, and 0.55) and at four different ages (7, 14, 28 and 96 days), using a rebound hammer (RH), a two-channel ultrasonic testing (UT) system, and a synthetic aperture radar (SAR) imaging system are presented. Our major findings are summarized in the following.

- Comparison of NDE methods: Among the three NDE methods used in this research, UT and RH are contact techniques requiring direct coupling with the surface of structures. Therefore, they are time-consuming and labor intensive. Only SAR is a non-contact technique capable of remote sensing. Since UT and RH directly interrogate the mechanical properties of concrete, they are more sensitive and accurate to the compressive strength estimation of concrete. While SAR indirectly interrogates the mechanical properties of concrete, the combined use of SAR with UT can improve the accuracy of UT alone, as well as enhancing inspection efficiency.
- Performance of individual NDE techniques – From our experimental data on predicting the compressive strength of concrete cylinders using three NDE techniques, the performance ranking among these three NDE techniques is (from the best) UT, SAR imaging, and RH. SAR imaging exhibited the best performance on predicting the early-age (7-day) compressive strength of concrete, while UT showed the best performance on the 14-day and the 96-day data. RH gave the best performance on the 28-day data. It should be noted that all predictions by three NDE techniques show a R^2^ value greater than 0.96122, demonstrating the overall good quality control in our laboratory experimentation. Our result indicated that transmissive mechanical sensing is more accurate than reflective mechanical sensing. However, it is also known that reflective mechanical sensing is more practical than transmissive mechanical sensing for in-situ applications.
- Performance of combined NDE techniques – When combining two NDE techniques to predict the compressive strength of concrete of different w/c ratios and at different ages, the best combination is SAR with UT, followed by SAR with RH and UT with RH. It is noteworthy to point out that the use of SAR improves the performance of either UT or RH, demonstrating the promising potential of multiphysical NDE.
- Selectivity in the combination of NDE techniques – When combining three NDE techniques, however, its performance is worse than the combination of two NDE techniques (SAR with UT). This result indicates the selectivity of multiphysical combination for NDE applications. Improved performance is selective to the nature of NDE technique. In our research, multiphysical NDE is superior to uniphysical NDE. Combinations of one EM technique with one mechanical NDE technique can improve the performance of all individual techniques. But combining two mechanical NDE techniques does not improve the performance of all individual techniques.

From our experimental result and data analysis on concrete specimens, we demonstrated the improvement of combined, multiphysical NDE techniques over individual NDE techniques on the prediction of concrete compressive strength, with the novelty to use SAR imaging in multiphysical NDE for the first time in the literature. Evidentially, it is shown that electromagnetic waves and mechanical waves are complementary to one another when it comes to understanding/assessing the condition of concrete. Meanwhile, more experimental datasets are needed before we can generalize the proposed empirical models to other w/c ratios and ages. Future research topics include the use of non-uniform weightings in data analysis.

## Data Availability

The datasets used and/or analysed during the current study are available from the corresponding author on reasonable request.

## References

[1] Mindess, S., Young, J. F. & Darwin, D. *Concrete* (Prentice-Hall, 2003).

[2] Mehta, P. K. & Monteiro, P. J. M. *Concrete: Microstructure, Properties, and Materials* (McGraw-Hill, 2005).

[3] Neville, A. M. *Properties of Concrete* (Prentice-Hall, 2012).

[4] Çolak, A. A new model for the estimation of compressive strength of Portland cement concrete. *Cem. Concr. Res.* **36**, 1409–1413. 10.1016/j.cemconres.2006.03.002 (2006).

[5] C39, Standard test method for compressive strength of cylindrical concrete specimens (ASTM, 2021) 10.1520/C0039_C0039M-21.

[6] C1074, Standard practice for estimating concrete strength by the maturity method (ASTM, 2019) 10.1520/ C1074-19.

[7] ACI 318, Building code requirements for structural concrete (American Concrete Institute, 2019) 10.14359/51716937.

[8] D6432, Standard guide for using the surface ground penetrating radar method for subsurface investigation (ASTM, 2019) 10.1520/D6432-19.

[9] T. Yu, A. Sinha, J. Wei, R. Bates, T. Dhant, H.N. Gandhi, (2021) Short-term mechanical strength prediction of ultra-high-performance concrete using noncontact synthetic aperture radar imaging. SPIE115921D, 10.1117/12.2584809

[10] Qasrawi, H. Y. Concrete strength by combined nondestructive methods Simply and reliably predicted. *Cement Concrete Res.* **30**, 739–746 (2000).

[11] Abo-Qudais, S. A. Effect of concrete mixing parameters on propagation of ultrasonic waves. *Constr. Build. Mater.* **19**, 257–326. 10.1016/j.conbuildmat.2004.07.022 (2005).

[12] Morris, I. M., Kumar, V. & Glisic, B. Predicting material properties of concrete from ground-penetrating radar attributes. *SAGE J. Struct. Health Monit.* **20**, 2791–2812. 10.1177/1475921720976999 (2021).

[13] Yang, H. et al. Deep learning-based X-ray computed tomography image reconstruction and prediction of compression behavior of 3D printed lattice structures. *J. Addit. Manuf.* **50**, 102774. 10.1016/j.addma.2022.102774 (2022).

[14] Bansal, T., Talakokula, V. & Sathujoda, P. Machine learning-based monitoring and predicting the compressive strength of different blended cementitious systems using embedded piezo-sensor data. *Measurement* **205**, 112204. 10.1016/j.measurment.2022.112204 (2022).

[15] Jena, T. et al. Deep learning neural networks for monitoring early-age concrete strength through a surface-bonded PZT sensor configuration. *Measurement* **241**, 115698. 10.1016/j.measurment.2024.115698 (2025).

[16] Bansal, T., Azam, A., Morwal, T., Talakokula, V. & Saravanan, T. Assessing cement paste strength evolution under curing: An experimental and numerical investigation through equivalent stiffness parameter identified by embedded piezo sensors. *Measurement* **241**, 0263–2241. 10.1016/j.measurment.2025.115713 (2025).

[17] Kouddane, B., Sbartaï, Z. M., Elachachi, S. M. & Lamdouar, N. New multi-objective optimization to evaluate the compressive strength and variability of concrete by combining non-destructive techniques. *J. Build. Eng.* **77**, 107526. 10.1016/j.jobe.2023.107526 (2023).

[18] Alzeyadi, A. & Yu, T. Moisture determination of concrete panel using SAR imaging and the K-R-I transform. *Constr. Build. Mater.* **184**, 351–360. 10.1016/j.conbuildmat.2018.06.209 (2018).

[19] Lai, W. L., Kou, S. C., Tsang, W. F. & Poon, C. S. Characterization of concrete properties from dielectric properties using ground penetrating radar. *Cement Concrete Res.* **39**, 687–695. 10.1016/j.cemconres.2009.05.004 (2009).

[20] Ferreira, R. M. & Jalali, S. NDT measurements for the prediction of 28-day compressive strength. *NDT & E Int.* **43**, 55–61. 10.1016/j.ndteint.2009.09.003 (2010).

[21] Shen, P., Lu, L., He, Y., Wang, F. & Hu, S. Hydration monitoring and strength prediction of cement-based materials based on the dielectric properties. *Constr. Build. Mater.* **126**, 55–61. 10.1016/j.conbuildmat.2016.09.030 (2016).

[22] Kumar, V., Morris, I. M., Lopez, S. A. & Glisic, B. Identifying spatial and temporal variations in concrete bridges with ground penetrating radar attributes, MDPI. *Remote Sens.*10.3390/rs13091846 (2021).

[23] Saleh, E. F., Tarawneh, A. N. & Katkhuda, H. N. A comprehensive evaluation of existing and new model-identification approaches for non-destructive concrete strength assessment. *Constr. Build. Mater.* **334**, 127447. 10.1016/j.conbuildmat.2022.127447 (2022).

[24] El-Mir, A. et al. Machine learning prediction of concrete compressive strength using rebound hammer test. *J. Build. Eng.* **64**, 105538. 10.1016/j.jobe.2022.105538 (2023).

[25] Pucinotti, R. Reinforced concrete structure: Nondestructive in situ strength assessment of concrete. *Constr. Build. Mater.* **75**, 331–334. 10.1016/j.conbuildmat.2014.11.023 (2015).

[26] Ali-Benyahia, K., Kenai, S., Ghrici, M., Sbartaï, Z. M. & Elachachi, S. M. Analysis of the accuracy of in-situ concrete characteristic compressive strength assessment in real structures using destructive and non-destructive testing methods. *Constr. Build. Mater.* **366**, 130161. 10.1016/j.conbuildmat.2022.130161 (2023).

[27] Yuva, Y. Low-strength concrete properties in existing structures using NDT and core test results. *J. Build. Eng.* **76**, 107281. 10.1016/j.jobe.2023.107281 (2023).

[28] Bensaber, A., Boudaoud, Z., Toubal Seghir, N., Czarnecki, S. & Sadowski, Ł. The assessment of concrete subjected to compressive and flexural preloading using nondestructive testing methods, correlation between concrete strength and combined method (SonReb). *Measurement* **222**, 113659. 10.1016/j.measurement.2023.113659 (2023).

[29] C192, Standard practice for making and curing concrete test specimens in the laboratory (ASTM, 2019) 10.1520/C0192_C0192M-19.

[30] C805, Standard test method for rebound number of hardened concrete (ASTM, 2018) 10.1520/C0805_C0805M-18.

[31] C597, Standard test method for ultrasonic pulse velocity through concrete (ASTM, 2022) 10.1520/C0597-22.

